# The impact of clinical research activities on communities in rural Africa: the development of the Clinical Research Unit of Nanoro (CRUN) in Burkina Faso

**DOI:** 10.1186/1475-2875-13-113

**Published:** 2014-03-22

**Authors:** Halidou Tinto, Innocent Valea, Hermann Sorgho, Marc Christian Tahita, Maminata Traore, Biébo Bihoun, Issa Guiraud, Hervé Kpoda, Jérémi Rouamba, Sayouba Ouédraogo, Palpouguini Lompo, Sandrine Yara, William Kabore, Jean-Bosco Ouédraogo, Robert Tinga Guiguemdé, Fred N Binka, Bernhards Ogutu

**Affiliations:** 1 Unité de Recherche Paludisme et Maladies Tropicales Négligées, Centre Muraz, Bobo-Dioulasso, Burkina Faso; 2 Institut de Recherche en Sciences de la Santé/Direction Régionale (IRSS/DRO), Bobo-Dioulasso, Burkina Faso; 3 Clinical Research Unit of Nanoro (IRSS-CRUN), Nanoro, Burkina Faso; 4 Institut Supérieur des Sciences de la Santé (INSSA), Bobo-Dioulasso, Burkina Faso; 5 INDEPTH-Network, Accra, Ghana

## Abstract

**Background:**

The opportunities for developing new drugs and vaccines for malaria control look brighter now than ten years ago. However, there are few places in sub-Saharan Africa with the necessary infrastructure and expertise to support such research in compliance to international standards of clinical research (ICH-GCP). The Clinical Research Unit of Nanoro (CRUN) was founded in 2008 to provide a much-needed GCP-compliant clinical trial platform for an imminent large-scale Phase 3 malaria vaccine trial. A dynamic approach was used that entailed developing the required infrastructure and human resources, while engaging local communities in the process as key stakeholders. This provided a better understanding and ownership of the research activities by the local population.

**Case description:**

Within five years (2008–2013), the CRUN set up a fully and well-equipped GCP-compliant clinical trial research facility, which enabled to attract 25 grants. The research team grew from ten health workers prior to 2008 to 254 in 2013. A Health and Demographic Surveillance System (HDSS), which covers a total population of about 60,000 people in 24 villages was set up in the district. The local community contributed to the development of the facility through the leadership of the king and the mayor of Nanoro. As a result of their active advocacy, the government extended the national electrical grid to the new research center, and later to the entire village. This produced a positive impact on the community’s quality of life. The quality of health care improved substantially, due to the creation of more elaborate clinical laboratory services and the acquisition of state-of-the-art equipment.

**Conclusion:**

Involving the community in the key steps of establishing the centre provided the foundation for what was to become the CRUN success story. This experience demonstrates that when clinical trials research sites are carefully developed and implemented, they can have a positive and powerful impact on local communities in resource-poor settings, well beyond the task of generating expected study data.

## Background

The epidemiology of malaria in sub-Saharan Africa is changing, with a decline in malaria-attributed deaths [1]. This is largely due to a significant increase in the financial commitment to the battle against malaria, followed by the implementation and scaling-up of effective interventions, such as artemisinin-based combination therapy (ACT), long-lasting insecticidal bed nets (LLIN), intermittent preventive treatment in pregnant women with SP (IPTp-SP) and, more recently, the seasonal malaria chemoprophylaxis (SMC). Considering the funds that are currently available to the malaria research community, the opportunities for the development of new drugs and vaccines for malaria control look brighter now than 10 years ago [2]. However, it is unclear if and how the downward trend in malaria cases and deaths can be maintained and what new tools are needed for the eventual elimination of malaria. Despite reports of dramatic declines in the burden of malaria in a number of countries [3], it remains stubbornly high in many areas, such as Burkina Faso. Elsewhere, a decline in malaria was followed by its reintroduction due to close proximity to areas of appreciable transmission [4]. In this context, it is extremely important to establish the safety, efficacy, and effectiveness of new interventions [2]. Yet, there are few places in sub-Saharan Africa with the necessary infrastructure and expertise to conduct such research in accordance to international standards (ICH-GCP). Moreover, very few African research centres have been able to maintain the required infrastructure and expertise over time, despite having conducted GCP-compliant clinical trials in the past. One reason is that the subsistence of the centres is often linked to one or only a few specific projects, which are time-bound and resource-limited. Once a specific project ends, experienced staff cannot be maintained. A number of centres in the region operate as field sites for Northern institutions. Therefore, they lack the leverage of autonomy to drive a local agenda and development plan. Such circumstances have major impacts on the ability of truly African centres, with competent local investigators, to compete for international funds. Nevertheless, it should be recognized that in the most recent years this is changing. In fact, some of African key senior scientists through some initiatives, such as the European and Developing Countries Trial Partnership (EDCTP) are becoming part of the main drivers of the research agenda.

There is a clear need to establish sustainable clinical research centres operating in accordance to international standards for the timely evaluation of new products and interventions [5]. This recognition led to the creation of the Clinical Research Unit of Nanoro (CRUN), which today provides a ready platform for testing new interventions in the environment where eventually they may be deployed. The CRUN is jointly run by two Burkinabe medical research institutions—namely the Centre Muraz (CM) and the Institut de Recherche en Sciences de la Santé (IRSS)—in collaboration with the Saint Camille Medical Center of Nanoro (CMA), which is a referral hospital for the District of Nanoro and is run by an Italian religious order in partnership with the government. The team was able to set-up a high-quality platform fully compliant with international standards for training and research and a Health and Demographic Surveillance System (HDSS) of about 60,000 people. To establish this capacity, CRUN leaders used a dynamic approach that engaged the local community throughout the process.

### The birth of CRUN

**Figure 1:**
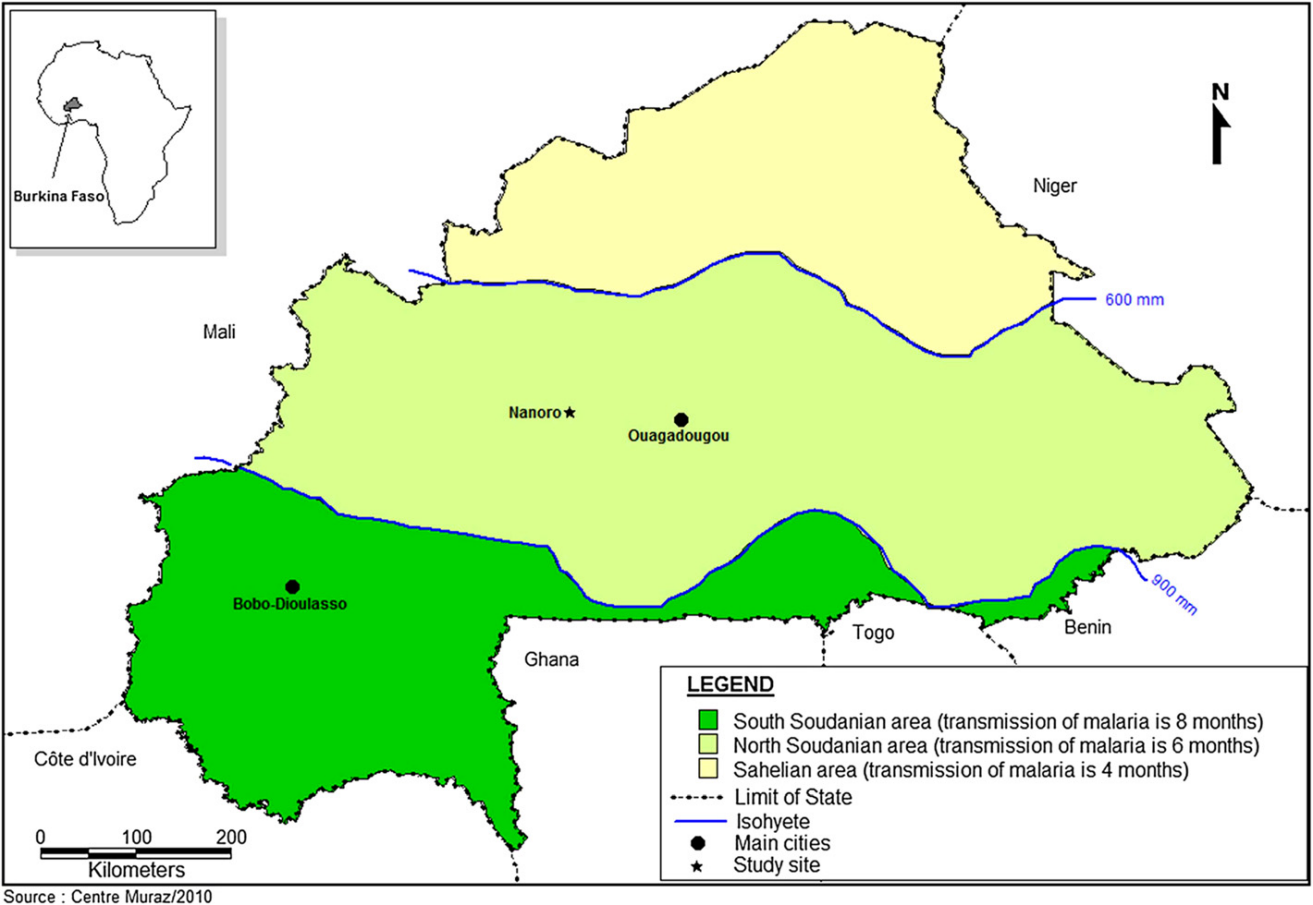
Map of Burkina Faso showing Nanoro and the three malaria transmission zones.

CRUN has been established by researchers from Centre Muraz and Institut de Recherche en Sciences de la Santé (IRSS), the two leading health researcher institutes in Burkina Faso. Centre Muraz (CM) entered into the malaria research field in 1985, when it implemented the continuous surveillance of malaria drug resistance in Burkina Faso. In the years that followed, this activity was carried out by CM in collaboration with IRSS and the National Malaria Control Programme (NMCP), after the creation of the latter in 1990 [6]. In 1998, the NMCP stratified the country into three zones according to their malaria transmission pattern (Figure 1). In each zone, two sentinel sites were established for the epidemiological surveillance of anti-malarial drug resistance in the framework of the Roll Back Malaria initiative [7]. The village of Nanoro was one of the two sentinel sites chosen in the central area. The health district of Nanoro is one of the five districts of centre-west health region. It is situated at approximately 90 Km from Ouagadougou, the capital city. In 2013, the total population was estimated at 158,127 people (data from the National Institute of Statistics and Demography). The population growth rate is 3.06% (2013 estimate). Mossi represent the main ethnic group, with the remaining represented by Gurunsi and Fulani. About 90% of the population is engaged in subsistence agriculture, which is vulnerable to periodic droughts. The literacy rate is low in both men and women (about 23%). The epidemiological profile of diseases remains dominated by communicable infectious diseases. The inadequacy of drinking water, individual and collective hygiene, associated to poverty is the main reasons of health problems. The general morbidity is high and malaria accounts for about 40% of the total burden of diseases. Maternal mortality rate is 300 deaths/100,000 live births (2010) and infant mortality rate is 78.3 deaths/1,000 live births. Malaria represents a significant burden on the population, but especially pregnant women and children; it is the first cause of consultation (35.12%), hospitalization (40.83%) and death (37.5%) (Ministry of Health [MoH]). As sentinel site, epidemiological studies on malaria were conducted in Nanoro beginning in 1998, including regular assessment of drug resistance. These studies were only carried out during the rainy season (six months per year). The sentinel site of Nanoro attracted more interest during 20012004, when resistance to chloroquine (at that time, the recommended first line treatment of uncomplicated malaria) emerged for the first time in the area. The data generated from these studies provided evidence that led the NMCP to change the national malaria treatment policy in 2005, from chloroquine to artemisinin-based combination therapy(ACT), namely artesunate-amodiaquine (ASAQ) and arthemeter-lumefantrine (AL) [8]. During this period, the research team did not reside in Nanoro, instead the team members regularly travelled to Nanoro to collect data and then returned to their respective institutions at the end of each study. With the historic detection of chloroquine resistance in the area, and the growing recognition of the need to establish more clinical research centres in Africa, the CRUN leaders proposed to establish a permanent team and facility in Nanoro. The proposal benefited from a favourable context of increasing political and financial support for malaria research. Although the site was disadvantaged by not being on the national electrical grid, the research team could successfully conduct its first clinical trial at ICH-GCP standards on ACT in 2005 [9]. In 2007, a second study on ACT, funded by the European and Developing Countries Partnership (EDCTP), was successfully conducted [10]. Based on this experience, the CRUN was selected in 2007 to be part of the RTS, S phase III malaria vaccine trial, a multicentre study at 11 centers in seven African countries [11]. As a major research contributor to the study, the CRUN joined the Malaria Clinical Trial Alliance (MCTA) network [5]. Technical support teams from MCTA in partnership with the PATH Malaria Vaccine Initiative (MVI) and GlaxoSmithKline (GSK Biologicals) visited the CRUN and identified the support required to execute the RTS,S phase III trial at Nanoro. The site was awarded infrastructure capacity enhancement grants by MCTA in 2007 and 2008 to refurbish the settings and upgrade the equipment. This support proved crucial for the successful execution of the RTS,S trial, and enhanced the site’s ability to conduct other trials to ICH-GCP standards. This track record led to the full establishment of the CRUN in 2008, followed by the inauguration of its new facility in April 2009 by the Minister of Health. A Health and Demographic Surveillance System (HDSS) was set up in the district. It covers a total population of about 60,000 people in 24 villages [12]. The HDSS was implemented according to INDEPTH guidelines, with regular surveys every four months and continuous monitoring by village reporters. This was enabled by being associate members of INDEPTH-Network. The CRUN researchers were able to access the INDEPTH resource materials and visit other INDEPTH member HDSS sites, while scientists from other HDSS sites on the network visited CRUN for technical support. The HDSS has become the bedrock of the CRUN research activities because it allows the identification, selection, and follow-up of study participants, as well as the monitoring of population and disease dynamics. CRUN is now a full member of the INDEPTH network and able to contribute datasets for joint analysis. This initiative was also supported by the Belgium cooperation through the Institute of Tropical Medicine (ITM) Framework Agreement 3 (FA3: 2008–2013), which has served as the main source of operational and training support since 2008.

The main objective of establishing the CRUN was to develop and maintain an appropriate expertise and infrastructure ensuring high quality clinical research and training. The vision of the CRUN is to become a prominent research and training centre in sub-Saharan Africa, to participate in the formulation of African public health strategies and programs, and to provide competent expertise in the health field. The CRUN’s mission is to provide evidence-based information for the health care of populations living in tropical countries, with a specific focus on malaria. This will be achieved by providing an excellent platform for training and research in tropical diseases in full compliance with international standards. This platform could be offered to address some specific research questions relevant to the MoH local agenda and development plan. In fact, having the Centre Muraz as partner in this initiative gives the opportunity to the CRUN to have a link with the MoH stakeholders such as the National Programs, including the malaria control programme and the national drug regulatory authorities, helping in the translation of research findings into practice.

### Organization and management

The two institutions (CM and IRSS) collaborating to run the CRUN are both located in Bobo-Dioulasso in the south-west of Burkina Faso. They are closely linked in and have been working together for the last 20 years. The CM is under the auspices of the Ministry of Health, while the IRSS is housed in the Ministry of Research and Innovation. The CM/IRSS is the largest medical research group in Burkina Faso and among the largest in the West African Francophone countries. The team’s scientific interest is mainly malaria research, but studies on other parasitic diseases (helminthiasis, filariasis, schistosomiasis) have been conducted. The team provides also support for training health practitioners (MD, PharmD, Msc, PhD) in collaboration with national universities (Ouagadougou and Bobo-Dioulasso). The linkage to academic institutions is a great asset as it provides a pathway for career development, sustainable career progression, and reliable availability of younger scientists.

At the inception of the research activities in Nanoro, the CRUN management structure was project oriented, i.e., each project had its own leader and team, with little coordination among the different projects. However, this changed in 2010 with the introduction of a more transversal organization across the different projects. The CRUN has been organized in five sections: laboratory, clinical activities, IT & data management, Health & Demographic Surveillance System (HDSS), and pharmacy, each with a designated manager.

### Achievements

#### *Infrastructure refurbishment and equipment upgrades*

Before the grants provided by MCTA (2005–2008), the CRUN was hosted by the CMA of Nanoro and research activities were conducted within the hospital complex. A small clinical ward was dedicated to clinical trial activities, while the laboratory facility was shared with the hospital. In 2009, the site constructed a new clinical trial facility, which was funded by MCTA, and where most of its research activities was transferred to. A fully functioning clinical laboratory (parasitology, biochemistry, haematology, microbiology and blood/cells culture) has been set up with the new equipment, including back-up equipment to ensure reliable services. The government of Burkina Faso, in recognizing the national interest in clinical research for a vaccine and other malaria interventions, extended the national electrical grid to the trial site and the village of Nanoro. Providing reliable electricity has proven to be a great achievement for this challenging initiative. Throughout the development of CRUN, the leadership of MCTA remained engaged, providing invaluable technical and mentorship support. A grant was provided in 2008 by the Belgium Cooperation through the Institute of Tropical Medicine Framework Agreement 3 (ITM FA3) was also of significant help. Although modest compared to MCTA funds, it provided support for three PhD grants and thus, contributed to creating a core group of young scientists able to attract additional new grants.

#### *Research activities and grants obtained*

Before CRUN was fully established, the site was only able to conduct two studies from 2005 to 2008. After obtaining the grants from MCTA in 2007 and 2008, and later with the grant from the Belgium cooperation (FA3), the centre was able to attract 25 grants from 2008 to 2013 (Tables 1 and 2). Similarly, the number of staff working at the CRUN increased from 10 people before 2008 to 254 people in 2013. This was possible due to the quality of the physical infrastructure and equipment available. Support from MCTA and MVI to set up a rigorous quality management system has also contributed to the success of the centre.

**Table 1:** List of research projects grants awarded since the CRUN establishment in 2008

**Year**	**Title of the study/activity funded**	**Funder**
2008	Pharmacovigilance for Artemisinin-based combination treatments in Africa	World Health Organization/(WHO/TDR)
	Multi-drug resistance in malaria under combination therapy Assessment of specific markers and development of innovative, rapid and simple diagnostics (MALACTRES)	European Union (FP7)
	Pharmacokinetics of Mefloquine-Artesunate in Pregnant Women with *Plasmodium falciparum* infection	Malaria in Pregnancy (MiP) Consortium
	A Phase II multicenter, efficacy and safety study of parenteral SAR97276A	Sanofi Aventis
2009	Safe and efficacious artemisinin-based combination treatments for African pregnant women with malaria	European and Developing Countries Partnership (EDCTP)
	Efficacy of GSK Biologicals. candidate malaria vaccine (257049)against malaria disease caused by *P. falciparum* infection in infants and children in Africa	Malaria Vaccine Initiative (MVI)
	Parallel group, double-blind, randomized study assessing the efficacy, safety and pharmacokinetic profile of ferroquine associated with artesunate	Sanofi aventis
2010	Assessment of the Safety of Anti-malarial Drug Use During Early Pregnancy	Malaria in Pregnancy (MiP) Consortium
	Malaria risk prior to and during early pregnancy in nulliparous women receiving long-term weekly iron and folic acid supplementation	National Institute of Health (NIH)
	Epidemiology study of malaria transmission intensity in sub-Saharan Africa	Malaria Vaccine Initiative (MVI)
	Study of immune correlates of protection against malaria after vaccination with RTS, S/AS01E	Malaria Vaccine Initiative (MVI)
	Multi-methods study on Clinical Trial participation and the informed consent process in vulnerable populations	Belgian cooperation
2011	Site characterization as a prelude to a study of malaria elimination using a combination of malaria control strategies in the Sahel region of Burkina Faso	Malaria Capacity Development Consortium (MCDC)
	Phase III randomized, open, controlled study to evaluate the immune response to the hepatitis B antigen of the RTS,S/AS01_E_ candidate vaccine	GlaxoSmithKline (GSK) Biologicals
	Improved quality of diagnostic services for malaria in pregnancy	World Health Organization/(WHO/TDR)
2012	Severe malaria and invasive bacterial disease among children: proportions, incidence rates and diagnostic value of Malaria Rapid Diagnostic Tests (ongoing)	Institute of Tropical Medicine (Belgium Cooperation)
	Community-based scheduled screening and treatment of malaria in pregnancy for improved maternal and infant health: a cluster-randomized trial in The Gambia, Burkina Faso and Benin	European Union (EU FP7)
	Genomic and environmental risk factors for cardio-metabolic disease in Africans.	National Institute of Health (NIH)
2013	Study of the Incidence, Reservoir and Routes of Transmission of Invasive Salmonellosis in Burkina Faso (IRTIS -BF)	Institute of tropical medicine (Belgium Cooperation)
	An open-label, single-arm study to evaluate the efficacy, safety and PK of Artemether-Lumefantrine dispersible tablet in the treatment of acute uncomplicated Plasmodium falciparum malaria in infants <5 kg body weight	Novartis
	A phase II, open-label, multicentre, pharmacokinetic, pharmacodynamics and safety study of a new paediatric eurartesim dispersible formulation and crushed film coated eurartesim tablet, in infant patients with Plasmodium falciparum malaria	Sigma Tau
	Observational study to evaluate the clinical safety after introduction of the fixed-dose Artemisinin-based Combination Therapy Eurartesim® (dihydroartemisinin/piperaquine [DHA/PQP]) in public health districts in Burkina Faso, Mozambique, Ghana and Tanzania	Indepth network

**Table 2:** List of other activities’ grants awarded since the CRUN establishment in 2008

**Year**	**Title of the study/activity funded**	**Funder**
2008	Grant to support the establishment of Malaria Clinical Research Unit in Burkina Faso	Belgian cooperation
	Support for the establishment of a Pharmacovigilance system for ACT safety monitoring in Burkina Faso	Sanofi aventis
2010	Good Clinical Practice: a theoretical and practical training in Burkina Faso	Belgian cooperation

CRUN’s enhanced infrastructure has enabled the centre to network with other research and academic centres, for example, by hosting two international GCP courses in 2010 and 2012, supported by Belgium Cooperation and the Institute of Tropical Medicine (ITM) Clinical Trial Strategic Network activities. The two courses brought together 34 participants from four different geographical areas (East and West Africa, Southeast Asia and Latin America) [13]. The course had a very innovative approach, consisting of theory, case studies and a practical segment in the field. Having participants from different geographical areas provided a dynamic experience as the participants examined clinical research challenges from diverse regions in a complementary manner.

### Impact of the CRUN establishment on the community

In the literature, few studies have been conducted to address the impact of clinical trials and clinical trial platforms on trial participants, their families and communities [14-16]. Lessons learnt from these reports indicate that clinical trials, especially when conducted in resource-poor settings, may negatively impact the quality of health care, e.g., by diverting resources from routine clinical activities, brain-drain among the best qualified health staff, discrimination of enrolled *versus* non-enrolled persons, or differentiating trial investigators from other health staff. The clinical trial may also have a negative impact on the relationship between a health facility and the community; for instance, in cases of poor or inappropriate communication about the nature and the objectives of the research or a lack of knowledge about local culture and customs by researchers. Conversely, clinical trials can contribute to the quality of routine healthcare and thus the wellbeing of a community, either directly (additional training of health staff and structural upgrades of health facilities) or indirectly (improved access to healthcare that improves the quality of routine healthcare [17].

No formal study has been conducted to assess the impact of the CRUN on the Nanoro community; however, the interactive approach led by local researchers obviated the negative perceptions and lack of understanding about the intentions and conduct of CRUN’s research activities. Researchers involved the community (district health management, elders, chief, and opinion leaders) in the founding steps of establishing the CRUN. To make the community a real partner in the centre’s activities, a tacit agreement was made that priority would be given to local people, in a competitive manner, for all non-professional jobs (construction workers, drivers, cleaners, field workers, data clerks, and others). Of the 254 people employed at the CRUN, about one-third come from Nanoro. This has strengthened the sense of ownership of the centre’s activities by the community. Through the modest creation of new jobs, CRUN makes a substantial contribution to reducing poverty in the community. In addition, staff members residing in Nanoro contribute to the micro-economy there.

Another crucial benefit for Nanoro and CRUN stemming from their productive engagement was electrification for the area. This was made possible by the mayor of Nanoro leading the negotiations for extending the national electrical grid to the CRUN, and with it, to the village of Nanoro. Electrification spurred a lot of economic activity and social amenities that enhance the wellbeing of the community, such as: (1) improved water supply through use electricity instead of generator; (2) ability to use electrical devices, such as fans during the hot season (when temperatures can reach 45-47°C), lighting so students can study at night, the use of refrigeration to safely store food and the extension of business hours past sunset.

Health care services have been improved through CRUN’s new microbiology laboratory. Before this laboratory was established, local patients had to travel about 100 km to the capital city, Ouagadougou, for the service. In addition, putting the local hospital on the electrical grid improved the working conditions of the health professionals, notably the ability to conduct surgery 24 hours a day and optimal storage of EPI vaccines at district health facilities. Last, but not least, in terms of benefits, is the installation of digital X-ray equipment (the first in Burkina Faso), which is now used for all patients attending the hospital regardless of their participation in the clinical trials.

## Conclusion

The innovative interactive approach to developing the CRUN based on active engagement with the community, have been central to establishing the centre and providing the foundation for the CRUN success story. Aside from the positive impact of the infrastructure upgrade on the standard of health care services, the electrification of the trial site positively impacted the quality of life in the community. This demonstrates that strong positive advocacy can lead African governments to take on more responsibility in supporting research activities. However the main challenge for CRUN in the future will be to secure and strengthen the capacity established. For that, the CRUN future strategic development plans should take into account the “epidemiological transition” of malaria with the decline of the number of clinical cases in endemic countries and the concomitant need for research in other endemic diseases. Therefore, beside malaria, currently the main research subject, the activity of the unit should be gradually expanded to other diseases, such as invasive bacterial infections, tuberculosis, HIV, reproductive health, schistosomiasis and other neglected diseases. In addition, the HDSS platform which is so far mainly used for research activities should be more and more used to guide the district health management team. Indeed, the HDSS by providing reliable health information such as burden of disease profiles will contribute to a better evaluation of health interventions by the Nanoro Health District. The success story of the CRUN establishment demonstrates that the impact of clinical trials on the community can be highly positive in resource-poor settings if the concerned community is involved in the process.

## Competing interest

HT, IV, HS, MCT, MT, BB, IG, HK, JR, SO, SY, WK, JBO, RTG are researchers affiliated to the Clinical Research Unit of Nanoro. FNB and BO are affiliated to the Indepth-Network.

## Authors’ contribution

HT, IV and HS drafted the manuscript. MCT, MT, BB, IG, HK, JR, SO, PL, SY, WK, JBO, RTG, FNB and BO corrected the manuscript and improved the content. All authors read and approved the final manuscript.
